# Neonatal Candidemia in Latin America: Trends, Resistance, and Prevention Strategies (2008–2025)

**DOI:** 10.3390/jof12030230

**Published:** 2026-03-23

**Authors:** Fredi Giovanni Soto Guzmán, Pilar Rivas-Pinedo, Jose Millan Onate Gutierrez

**Affiliations:** 1 Department of Microbiology, Faculty of Medicine, Universidad Nacional de Colombia, Bogotá 111321, Colombia; 2 Medical and Diagnostic Mycology Group, Department of Microbiology, Faculty of Medicine, Universidad Nacional de Colombia, Bogotá 111321, Colombia; 3 One Health Research Group for Infectious Diseases, Keralty, Department of Internal Medicine, Clínica Colsanitas S.A., Clínica Sebastián de Belalcázar, Cali 760045, Colombia; 4 Infectious Diseases Service, Clínica Imbanaco, Cali 760042, Colombia; 5 Infectious Diseases Service, Clínica de Occidente S.A., Cali 760035, Colombia

**Keywords:** neonatal candidemia, invasive candidiasis, neonatal intensive care units, Latin America, *Candida parapsilosis sensu lato* (s.I.), *Candidozyma auris*, antifungal resistance, antifungal stewardship, premature infant, fluconazole prophylaxis

## Abstract

Candidemia and invasive candidiasis remain significant causes of late-onset sepsis and mortality in very-low-birth-weight infants, especially in low- and middle-income countries. This narrative review synthesizes studies published between 2008 and 2025 in Latin America, addressing epidemiology, species distribution, antifungal susceptibility patterns, risk factors, therapeutic approaches, and clinical outcomes, with international comparisons. Accordingly, we present a qualitative narrative synthesis (see Methods) rather than a formal year-over-year temporal trend quantification. Globally, five species predominate, namely *Candida albicans*, *C. parapsilosis sensu lato* (s.I.), *Candida tropicalis*, *Nakaseomyces glabratus*, and *Pichia kudriavzevii*, with a sustained increase in non-albicans species and growing resistance to fluconazole. In Latin America, the burden varies depending on the hospital setting; *C. parapsilosis sensu lato* (s.I.) predominates in NICUs, and *Candidozyma auris* has emerged, associated with nosocomial outbreaks and multidrug resistance. Factors such as extreme prematurity, prolonged catheter use, parenteral nutrition, and antibiotics are consistently associated with the risk of infection. Mortality remains high, influenced by diagnostic delays and species characteristics. Standardized microbiological surveillance, accurate identification, and strategies tailored to each clinical setting are required to improve outcomes in this vulnerable population.

## 1. Introduction

Neonatal candidemia continues to be a significant cause of late-onset sepsis (LOS), with important implications in terms of morbidity and mortality and risk of neurodevelopmental disorders, particularly in newborns with very low or extremely low birth weight (VLBW/ELBW). These patients have significant immunological and physiological vulnerability: the immaturity of the immune system, the fragility of the mucocutaneous barriers, early colonization by *Candida* spp., and the frequent need for invasive interventions—such as the use of central venous catheters (CVCs), mechanical ventilation (MV), or total parenteral nutrition (TPN)—create fertile ground for the development of fungal infections. Globally, both population registries and active surveillance systems agree that candidemia and invasive candidiasis (IC) in neonates occur mainly in highly complex clinical settings. Incidence rates may vary according to the criteria used—live births (LB), hospital admissions, or episodes of sepsis—but the associated mortality rate exceeds 20% in many cases, with a particularly high impact in low- and middle-income countries (LMICs) [1,2,3,4,5,6,7,8].

In this scenario, the etiological distribution of neonatal candidemia has undergone notable changes in recent decades. Although *Candida albicans* remains the most frequently identified species in many centers, there has been a progressive shift toward non-*albicans* species, such as *C. parapsilosis sensu lato* (s.I.), *Candida tropicalis*, *Nakaseomyces glabratus* (formerly *Candida glabrata*), and *Pichia kudriavzevii* (formerly *Candida krusei*), which have more variable and, often, less predictable antifungal susceptibility profiles. Surveillance systems, both globally and regionally, have documented a relative decline in the frequency of *C. albicans*, accompanied by a sustained increase in the prevalence of non-*albicans* species and the emergence of resistance patterns, particularly to fluconazole (FCZ) and, to a lesser extent, to echinocandins. These trends, however, are not homogeneous and show significant geographical differences, highlighting the importance of analyzing microbiological findings within the local epidemiological context in order to make informed and contextualized therapeutic decisions [2,3,4,5,6,9,10,11].

In Latin America, the burden of neonatal candidemia shows remarkable heterogeneity, reflecting marked differences in access to therapeutic resources, diagnostic capacity, and epidemiological surveillance systems. Data collected through multicenter studies and national registries show variable incidence rates, ranging from relatively low figures per 1000 LB to considerably higher proportions in high-risk neonatal subgroups. In neonatal intensive care units (NICUs), *C. parapsilosis sensu lato* (s.I.) is usually the predominant species, frequently associated with the use of invasive devices. Because most regional reports do not routinely resolve the *C. parapsilosis* complex to the species level, we use this term unless sensu stricto identification is explicitly reported. Likewise, nosocomial outbreaks linked to horizontal transmission and biofilm formation have been reported, adding complexity to the control of these infections and the implementation of effective preventive strategies [5,9,12,13,14,15,16,17,18,19,20,21,22,23,24,25,26]. Adding to this scenario is the growing concern about the emergence of *Candidozyma auris* (formerly *Candida auris*), recently classified by the World Health Organization (WHO) as a priority fungal pathogen. This species is distinguished by its multidrug resistance to various types of antifungal agents, the difficulties in its microbiological identification, and its ability to cause persistent nosocomial outbreaks. Its impact on NICUs has been significant, with reports in pediatric and neonatal populations in several countries in the region, underscoring the urgent need for specific surveillance, infection control, and rapid response upon detection [21,27,28,29,30,31,32,33,34,35,36,37,38].

The combination of clinical vulnerability in newborns, changes observed in the ecology of *Candida* spp., the emergence of new patterns of antifungal resistance, and persistent deficiencies in infection prevention and control (IPC) strategies creates a particularly complex scenario for NICUs in Latin America. In this context, therapeutic decision-making requires a comprehensive assessment that considers key factors such as the isolated species, minimum inhibitory concentration (MIC), gestational age of the patient, and local epidemiological profile. These considerations inform the practical priorities for prevention and IPC discussed later, including safe and standardized catheter/TPN management, rational antimicrobial use, and strengthened active microbiological surveillance with species-level identification.

This review article, covering the period 2008–2025, presents an updated summary of the epidemiology of candidemia and IC in neonates in Latin America, in dialogue with information from international references, including the United States, Europe, and surveillance networks in LMICs. Importantly, the regional literature remains heterogeneous and frequently limited to single-center cohorts, outbreak reports, or short surveillance snapshots, which have limited comparability across studies and the translation of findings into actionable prevention priorities across Latin American NICUs. It describes the most commonly implicated fungal species, their antifungal susceptibility profiles, the main risk factors, and the most relevant clinical outcomes. In addition, the clinical and epidemiological implications of the emergence of *C. auris* and other multidrug-resistant pathogens in the neonatal setting are examined. Based on this critical analysis, priority lines of action are proposed aimed at strengthening microbiological surveillance, improving the efficacy of antifungal treatment, and consolidating sustainable IPC policies in NICUs in the region [1,2,3,4,5,6,7,8,9,10,11,12,13,14,15,16,18,19,20,21,22,27,28].

This manuscript was conceived and developed as a narrative review. As this is a narrative review with qualitative synthesis (and not a systematic review), PRISMA 2020 reporting guidelines are not applicable; however, we provide a transparent description of the databases searched, key terms, eligibility criteria, screening approach, and the date of the last update. To further support methodological quality and transparency appropriate for narrative reviews, we considered the SANRA domains (importance, clear aims, search description, adequate referencing, scientific reasoning, and appropriate presentation of relevant data). To build a clear and practice-oriented synthesis, we searched the biomedical literature published between 2008 and 2025 on neonatal candidemia/IC in Latin America using major databases (PubMed/MEDLINE, Embase, Scopus, Web of Science Core Collection, LILACS, and SciELO) and by screening the reference lists of key papers. To contextualize regional findings, we also included selected international surveillance reports and studies from high-income settings (Europe and North America) and other LMICs when they provided relevant benchmarks for species distribution, antifungal resistance, and prevention strategies. Our search combined controlled vocabulary and free-text terms reflecting the core concepts of the review, including “neonatal candidemia”, “invasive candidiasis”, “neonatal intensive care unit/NICU”, “Latin America” (and individual country names), “*Candida parapsilosis sensu lato* (s.l.)”, “*Candidozyma auris*”, “antifungal resistance”, “antifungal stewardship”, “premature infant”, and “fluconazole prophylaxis.” Search strings were adapted to each database’s syntax and indexing (e.g., MeSH terms in PubMed/MEDLINE and Emtree terms in Embase) and combined concepts for neonates/NICU, candidemia/invasive candidiasis, and Latin America (including country names). We included studies and reports from Latin American settings that provided extractable neonatal data (NICU or comparable high-acuity neonatal care), including observational cohorts/series, surveillance reports, and outbreak investigations. We excluded publications that fell outside the prespecified time window, studies not focused on neonates, reports not conducted in Latin America, duplicate datasets, and papers that lacked extractable information on neonatal candidemia or IC. Records were screened for relevance by the author team, and any uncertainties were resolved by consensus. Accordingly, findings are summarized using qualitative narrative synthesis rather than a formal meta-analysis or year-over-year trend quantification. The literature search was last updated on 8 December 2025.

## 2. Neonatal Candidemia in Latin America (2008–2025)

Neonatal candidemia remains an important cause of LOS, with a particularly high burden in preterm and VLBW newborns. In Latin America, both the magnitude of the problem and the ecology of *Candida* spp. vary considerably between countries, institutions, and levels of care complexity, which directly influences the empirical selection of antifungal agents and the design of specific preventive strategies [5,6,9,12,13,15,16,19,20,23,24,25,26,39,40,41,42]. Across the multicountry Latin American surveillance summary (7 countries), C. albicans accounted for 37.6% and *C. parapsilosis sensu lato* (s.I.) for 26.5% of pediatric/neonatal bloodstream isolates [19]. In the Latin American NICU and country-level reports summarized in Table 1, these two species are repeatedly highlighted among the leading agents, although the dominant profile varies by setting and study. Beyond these, a sustained increase in non-albicans species has been documented, including *C. tropicalis*, *N. glabratus*, and other related phylogenetic complexes, many of which show variable susceptibility profiles to azoles and polyenes [6,9,13,15,16,19,23,24,26,39,41,42]. The detection of *C. auris* in NICUs in Colombia and Venezuela poses an additional challenge, not only because of multidrug resistance but also because of its capacity for hospital dissemination [21,27,28,30,31,32,33,34,35,37].

Across international neonatal cohorts, bloodstream infection due to *Candida* is repeatedly associated with device-intensive NICU care and the propensity of several yeasts to adhere to biomaterials and form biofilms on CVCs, endotracheal tubes, and other medical materials [1,3,4,5,6,8,18,43,44,45]. Within this context, many studies describe an evolving etiologic profile in which non-*albicans* species contribute an increasing share of cases and, in some settings, are more likely to display reduced susceptibility to azoles [1,3,4,5,6,8,18,43,44,45]. Prognosis is shaped by both host and care factors—especially gestational age, comorbidities, and delays in initiating effective therapy—as well as by the infecting species, with poorer outcomes reported among extremely premature infants and infections caused by intrinsically or multidrug-resistant organisms [1,3,4,5,6,7,8,18,43,45]. Consistent with these observations, international guidance underscores risk stratification, rigorous management of CVCs and other invasive devices, and empiric antifungal choices guided by local ecology and available susceptibility data [2,3,4,5,6,7,8,13,18,25,43,45,46,47,48].

The available evidence shows wide variability in neonatal bloodstream infection rates across Latin American settings. For example, the multicountry Latin American summary reported an overall incidence of 1.18 per 1000 admissions, with country estimates ranging from 0.33 (Chile) to 1.96 (Colombia) per 1000 admissions [19], while Mexico reported 2.27 per 1000 live births [16]. This variation is conditioned by the methodological design of the studies, the type of population analyzed—whether all newborns, only neonates with VLBW, or only patients treated in referral NICUs—and the surveillance system used. In Colombia, for example, a cohort of newborns hospitalized in a university center reported a frequency of *Candida* fungemia of 0.06%, with a predominance of *C. albicans* and *C. parapsilosis sensu lato* (s.I.) [14]. In Mexico, a hospital documented an incidence of invasive fungal disease (IFD) caused by *Candida* of 2.27 per 1000 LB, mainly in VLBW neonates with prolonged hospital stays [16]. In Brazil, a NICU multicenter report found candidemia in 10.97% of neonates with suspected sepsis, supporting *Candida* as a relevant LOS pathogen in high-risk settings [15,17,49,50]. In most of these studies, the median age at diagnosis is between 10 and 16 days of life, consistent with the clinical pattern of LOS, associated with cumulative exposure to invasive devices and antibiotics [6,12,13,16,23,24,25,26,39,51,52].

The risk of neonatal candidemia is mainly concentrated in medium- and high-complexity NICUs, driven by a set of well-established risk factors: extreme prematurity, VLBW, presence and duration of CVC, prolonged use of TPN and lipid emulsions, repeated or extended broad-spectrum antibiotic treatments, and previous colonization by *Candida* spp. [2,3,4,5,6,9,12,15,18,23,24,26,39,43,45,47]. In Latin America, *C. parapsilosis sensu lato* (s.I.) predominates in NICUs, with a significant role in outbreaks associated with horizontal transmission mediated by invasive devices or contact with healthcare workers’ hands, as well as the emergence of FCZ-resistant strains. In addition, the emergence of *C. auris* has been documented in some specific settings in the region [5,9,12,15,19,21,27,28,30,31,32,33,34,37,41,53,54]. The identification of environmental reservoirs, colonization of healthcare personnel, and the ability of these yeasts to form dense biofilms on plastic surfaces reinforce the critical role of horizontal transmission, underscoring the urgent need to strengthen infection control strategies in Latin American NICUs [5,9,11,41,53,55,56].

Multicenter pediatric surveillance in Latin America highlights that a substantial share of *Candida* bloodstream infections occurs in neonates and consistently points to *C. parapsilosis sensu lato* (s.I.) as a leading non-albicans species in NICU settings [9,12,20,25,40,41]. The NICU series from Brazil and Mexico further support this profile and report subregional variability in antifungal susceptibility, including documentation of isolates with fluconazole resistance and variable susceptibility to other azoles and polyenes [9,14,15,16,17,41,49]. These findings reinforce the need for setting-specific surveillance and susceptibility-informed therapeutic decisions.

In LMICs, recent data from the NeoOBS study and a contemporary meta-analysis have shown high rates of resistance to FCZ, as well as a significant proportion of *Candida* spp. isolates with reduced sensitivity to echinocandins in neonatal populations [5,6,9,10,11]. These findings are essential for contextualizing empirical therapeutic decisions and for appropriately adjusting clinical guideline recommendations in highly vulnerable settings. In particular, outbreaks caused by FCZ-resistant *C. parapsilosis sensu lato* (s.I.) have shown clonal and persistent patterns, which have direct implications for infection control strategies. These situations require, among other measures, intensive environmental interventions and careful selection of initial antifungal treatment [9,11,18,41,44,53,54].

Colombia reflects the regional pattern observed across multiple Latin American NICU reports, characterized by the predominance of *C. albicans* and *C. parapsilosis sensu lato* (s.I.), alongside an increasing contribution of non-albicans species and the added challenge of *C. auris*–associated outbreaks in selected settings [19,20,21,28,30,40,56]. These complex scenarios require a rigorous diagnostic and therapeutic approach, including accurate species identification, antifungal susceptibility testing (AFST), strict implementation of contact isolation, environmental disinfection with agents effective against multidrug-resistant yeasts, and antifungal selection tailored to both the susceptibility profile and the gestational age of the patient. These interventions should be aligned with international clinical guidelines and updated regional guidelines for the management of neonatal candidemia [2,3,5,9,11,12,22,31,34,43,45,46,47,48].

### 2.1. Global and Latin American Scenario

Globally, a meta-analysis conducted in LMICs between 2008 and 2022 estimated a pooled incidence of neonatal IC of 2.6% (95% CI: 2.2–3.0), with an overall case fatality rate of 18.7% and notable regional variation, reflecting differences in hospital infrastructure, access to NICUs, and prevention practices implemented [6]. The NeoOBS study (2018–2021), which brought together a multicenter cohort in LMICs, allowed for the contextualization of local rates—although not always standardized by site—and comparison of both species distribution and initial and targeted antifungal strategies, highlighting the high burden of *Candida* in cases of neonatal LOS [5,6]. In high-income countries (HICs), active surveillance by the CDC (Centers for Disease Control and Prevention) in four regions of the United States between 2009 and 2015 showed a decline in the incidence of neonatal candidemia from 31.5 to about 11 cases per 100,000 LB, with stabilization between 2012 and 2015, suggesting the positive effect of preventive strategies, targeted prophylaxis, and improvements in catheter management [7]. Meanwhile, the EUROCANDY study—conducted in 10 European countries between 2005 and 2015—represents one of the largest multinational series on pediatric candidemia, with nearly 36% of the population corresponding to the neonatal period. Although it does not report standardized rates at the continental level, it is a relevant source on species distribution and outcomes in high-income settings [8].

In Latin America, the epidemiology of neonatal candidemia shows considerable heterogeneity in reported rates, largely attributable to differences in the study design, the populations evaluated—whether all LB, VLBW newborns, or newborns admitted to the NICU—and the denominators used to calculate incidence (LB, hospital admissions, episodes of sepsis, confirmed BSIs, or healthcare-associated infections [HAIs]). Most of the available evidence focuses on the period between 2008 and 2019. The Latin American Candidemia Network, which included 23 hospitals in eight countries between 2008 and 2010, reported a pediatric incidence of 0.81 per 1000 admissions, with approximately 29% of cases occurring in the neonatal period, which is a key reference point for comparing subsequent cohorts [12,19]. Among the most recent data, those from Brazil stand out, where studies in high-risk NICUs describe IFD prevalences close to 10–11% among HAIs, and from Mexico, where an incidence of 2.27 per 1000 LB has been estimated in a reference center for IC [9,15,16,17,54]. In Colombia, multicenter studies have documented a national burden of candidemia higher than that observed in HICs, with a notable contribution from non-*albicans* species and *C. auris* in the pediatric population. However, significant gaps remain regarding specific neonatal estimates at the national level [13,19,20,21,28,30,40].

In Latin America, *Candida* spp. infection rates in newborns vary significantly depending on the denominator used. When expressed in terms of LB, the figures tend to be relatively low. In Bogotá, for example, an incidence of 0.62 per 1000 LB (8 cases in 12,905 LB) was reported for neonatal fungemia [15], while in Guadalajara, during the period 2015–2019, an incidence of 2.27 per 1000 LB was documented for IC [16]. In Lima, a study on LOS in NICUs reported 10.04 episodes per 1000 LB, with a predominance of bacterial etiology, although without a specific rate of candidemia expressed per LB, which limits its direct comparability in the context of fungal infections [51]. In high-risk “internal” populations, the proportions are considerably higher: in Pernambuco, Brazil, candidemia was detected in 10.97% of newborns with suspected sepsis (44 out of 401 cases) [15]; and in a national HAI surveillance study of high-risk neonates, 19 of 221 infections (8.6%) were fungal among 881 newborns, with a mortality risk approximately 2.6 times higher than that observed in bacterial infections [17]. These differences underscore the need to clearly specify the denominator used—LB, newborns evaluated for sepsis, confirmed BSI, or total HAIs—to enable valid comparisons between centers and countries, as highlighted in the most recent neonatal reviews and prevention guidelines [1,5,6,39,43,47].

Species distribution across Latin American NICU series is summarized in Table 1 and Table 2: *C. albicans* remains the most frequently reported species in most reports (≈31.8–60% of neonatal isolates), whereas Brazilian NICU cohorts consistently report *C. parapsilosis sensu lato* (s.I.) as predominant (≈38–40%) [9,13,14,15,16,17,19,20,23,24,25,41,42,55]. In addition, *C. auris* outbreaks reported in Venezuela and Colombia (2012–2017) were characterized by high azole resistance, variable amphotericin B (AmB) susceptibility, and outbreak-associated neonatal mortality approaching 40%, reinforcing the need for accurate species identification, routine AFST, and stringent NICU infection control [21,27,28,31,32,33,34,35,37].

Table 1 presents a summary of the burden of candidemia and IC with neonatal involvement in different Latin American countries, contrasting these data with reports from the main international surveillance networks. Although the rates are not directly comparable due to differences in the denominators used and the methodological approaches of each study, the analysis allows us to identify two distinctive patterns in the region: the relative predominance of *C. parapsilosis sensu lato* (s.I.) and the emergence of *C. auris*. Furthermore, resistance to FCZ shows considerable variability between countries and centers, and high MICs against echinocandins have already been reported in *C. parapsilosis sensu lato* (s.I.) isolates. These findings have direct implications for both the empirical selection of antifungals and the design of prophylaxis strategies in neonates [5,6,9,10,11,15,19,23,24,26,42,44]. This pattern is supported by the Latin American evidence compiled in Table 1 and Table 2, which include a multicountry regional surveillance report and multiple country-/NICU-level reports spanning 2008–2025.

### 2.2. Etiological Distribution of Neonatal Candidemia

The distribution of species in neonatal candidemia remains relatively stable in Latin America. *Candida albicans* continues to be the most frequently isolated species in most series, with proportions ranging from 31.8% to 60%, except in Brazil, where *C. parapsilosis sensu lato* (s.I.) shows sustained predominance, with frequencies close to 38.6% [9,14,15,16,17,22,23,24,26,42,55]. At the regional level, there has been a growing trend toward non-*albicans* species, along with the emergence of *C. auris*, a fungal pathogen classified as a priority due to its multidrug resistance, the difficulties in detecting it using conventional methods, and its ability to cause hospital outbreaks with high neonatal mortality. Between 2012 and 2017, outbreaks of *C. auris* were documented in Venezuela and Colombia, with resistance to FCZ and VCZ, variable sensitivity to Amphotericin B deoxycholate (d-AmB), and a mortality rate of close to 40%, reinforcing the need for specific species identification and systematic AFST in isolates from NICUs [21,27,28,30,31,32,34,35,49,51]. At the same time, other non-*albicans* species such as *C. parapsilosis sensu lato* (s.I.), *Meyerozyma guilliermondii* (formerly *Candida guilliermondii*), and *C. tropicalis* have increased in frequency in these units, closely related to the intensive use of CVCs, TPN, and other invasive devices [9,14,15,16,17,22,23,24,25,26,42,55].

Globally, more than 90% of CI episodes are concentrated in five species: *C. albicans, C. parapsilosis sensu lato* (s.I.), *C. tropicalis*, *N. glabratus*, and *P. kudriavzevii* [2,3,4,9,10]. In LMICs, a review that included more than 10,000 fungal isolates estimated that *C. albicans* accounted for around 39% of cases and *C. parapsilosis sensu lato* (s.I.) for 25%, with notable regional heterogeneity and a growing proportion of emerging and resistant species [6,9,10,11]. The neonatal substudy of the NeoOBS project (2018–2021), conducted in 14 hospitals in eight countries—including Brazil—confirmed the trend toward a progressive shift toward non-*albicans* species in NICUs. In this cohort, *C. albicans* accounted for 35% of bloodstream isolates, *C. parapsilosis sensu lato* (s.I.) for 30%, and *C. auris* for 14%. In addition, 59% of *C. parapsilosis sensu lato* (s.I.) isolates were found to be resistant to FCZ, a highly relevant finding for guiding empirical therapy in neonates at high risk of candidemia [5,6].

In Latin America, multicenter pediatric surveillance conducted between 2008 and 2010 already showed, in the neonatal subgroup, a mixed pattern in the distribution of species, consistent with high exposure to invasive devices and the NICU environment. During that period, *C. albicans* accounted for 43.8% of isolates, *C. parapsilosis sensu lato* (s.I.) for 27.0%, and *C. tropicalis* for 14.6%, with other species accounting for a smaller proportion [9,12,19]. More recent studies in Brazil have confirmed the predominance of *C. parapsilosis sensu lato* (s.I.) (complex) and *C. albicans* but have also revealed antifungal susceptibility profiles that call into question the efficacy of empirical treatment with FCZ, especially in contexts where the circulation of resistant strains of *C. parapsilosis sensu lato* (s.I.) has been documented [9,10,11,15,44,55]. In these scenarios, MICs for echinocandins tend to be higher than those observed for *C. albicans* or other species, making it necessary to carefully analyze therapeutic escalation based on clinical and epidemiological interpretation of the sensitivity data.

In Colombia, two clinical series conducted in pediatric and neonatal populations have documented the emergence of *C. auris*, associated with adverse outcomes and outbreaks in NICUs, highlighting the persistent challenges surrounding etiological identification, effective outbreak control, and targeted antifungal management, especially in resource-limited settings [20,25,28,37,56]. At the regional level, *C. auris* is no longer a sporadic finding but has become a priority threat to public health. In response to this situation, the Pan American Health Organization (PAHO) issued an epidemiological alert that explicitly included outbreaks in pediatric and neonatal populations, establishing a framework to strengthen species-specific surveillance and promote the early implementation of infection control measures in NICUs [11,31,35,36,38].

### 2.3. Antifungal Treatment in the NICU and Interpretation of Sensitivity Patterns

Therapeutic decision-making in NICUs requires careful interpretation of AFST results, considering both clinical cut-off points (ECV) and epidemiological values (ECOFF). This information must be integrated with the identified etiological agent, MIC, and clinical status of the patient to appropriately guide the initiation, escalation, or de-escalation of antifungal treatment [2]. Distribution by species directly influences the antifungal susceptibility profile, which can vary significantly between countries and over time. Therefore, local epidemiology is key both for defining the initiation of empirical treatment and for appropriately adjusting targeted treatment [3,4,5,9,10,12,15,19,25,41]. Table 1 summarizes the distribution by species of candidemia and neonatal IC in Latin America between 2005 and 2025, in contrast with international reference data (HIC, LMIC/NeoOBS, EUROCANDY). It includes the proportions observed in NICUs, the shift toward non-*albicans* species, and the emergence of *C. auris*, as well as, when available, information on antifungal susceptibility with a direct clinical impact on the choice of empirical treatment [5,6,7,8,9,10,11,19].

Available evidence in neonates from LMICs demonstrates considerable selective pressure on azoles. In a contemporary cohort from the NeoOBS study, aggregate resistance to FCZ reached approximately 40%, while no resistance to micafungin (MCF) was documented, highlighting a geographic concentration of resistant *C. parapsilosis sensu lato* (s.I.) isolates [5,6,10]. This pattern raises the need for caution in the empirical use of azoles and supports the initial choice of uncompromised agents, at least until the causative species and its MIC have been identified. A neonatal meta-analysis reinforces these observations, pointing to the lower reliability of azoles and the high overall activity of echinocandins, with the already known exception of clinical isolates of *C. parapsilosis sensu lato* (s.I.) [10]. In Latin America, a multicenter survey (2008–2010) reported a low overall rate of resistance to FCZ and echinocandins, although *C. parapsilosis sensu lato* (s.I.) predominated, a species that is intrinsically less sensitive to echinocandins and frequently associated with the use of invasive devices and horizontal transmission [9,19,41]. The latest data from Brazil have confirmed high MICs for echinocandins in isolates of *C. parapsilosis sensu lato* (s.I.) and *N. glabratus*, as well as the presence of strains of the *C. haemulonii* complex with high MICs for both FCZ and d-AmB, which complicates therapeutic decision-making when de-escalating antifungal treatment [9,10,11,15]. Regional pediatric surveillance supports that, once the infectious focus has been controlled and clinical stability has been achieved, de-escalation to FCZ should only be considered if the isolate is susceptible and the species involved does not present intrinsic or acquired resistance [12,22,40,43,45,47].

In Colombia, recent series have reported high sensitivity to FCZ and anidulafungin (ANF) in most *Candida* isolates, although with reduced sensitivity in *N. glabratus*, emphasizing the need to confirm the MIC before considering de-escalation of antifungal treatment [9,20,40]. In the pediatric population, *C. auris* has been consistently associated with low sensitivity to azoles and variable MICs against d-AmB, making therapeutic decision-making a process that requires systematic AFST. At the same time, strict implementation of infection control measures in NICUs is required to contain outbreaks and reduce mortality associated with this emerging species [21,28,30,31,33,34,35,38].

Overall, the greatest clinical risk in neonates is concentrated in settings where high rates of FCZ-resistant *C. parapsilosis sensu lato* (s.I.), multidrug-resistant *C. auris* profiles, and species with high MICs against echinocandins or d-AmB are identified [2,5,9,10,11,12,15,19,21,25,40,41,46]. The therapeutic implications for NICUs can be summarized as follows:

1. *Initiation of empirical antifungal treatment:* in settings with documented azole resistance, or when *N. glabratus* or *C. auris* is suspected, initiate an echinocandin or d-AmB. Selection should consider gestational age, neonatal PK, and the clinical syndrome, and be promptly reassessed once species identification and MICs are available [2,3,4,5,6,9,10,41,43,45,46,47,48].
2. *Therapeutic de-escalation:* step down to FCZ only when the isolate is susceptible, the infant is clinically stable, and source control has been achieved (e.g., CVC removal/replacement, drainage). FCZ should not be used for *P. kudriavzevii* (intrinsic resistance) and requires caution for *N. glabratus* (frequent DDS) [2,3,4,9,10,46].
3. *Species-specific considerations:*
  - *Candida parapsilosis sensu lato* (s.I.): monitor echinocandin MICs; consider FCZ de-escalation only if susceptible, clinically stable, and with adequate source control, avoiding outbreak settings with documented resistance [5,9,10,15,19,41].
  - *Nakaseomyces glabratus*: prioritize echinocandins; switch to high-exposure azoles only with favorable MICs and supportive clinical response [2,3,4,9,10,46].
  - *Pichia kudriavzevii*: prefer echinocandins or d-AmB; avoid azole monotherapy [2,3,4,9,10,46].
  - *Candidozyma auris*: individualize based on local susceptibility; echinocandins are generally first-line, alongside strict IPC to limit spread [11,21,28,30,31,32,33,34,35,37,38].
  - *Candida haemulonii* complex: confirm identification (ideally MALDI-TOF and/or molecular methods) given high MICs to d-AmB and FCZ; tailor therapy with microbiology/ID input [9,11,15,21].
4. *Laboratory:* request AFST systematically using validated methods; interpret results with ECVs/ECOFFs and ensure species-level identification. Timely lab–NICU communication is essential for early optimization and monitoring of local *Candida* ecology [2,9,10,46].
5. *Practice in the NICU:* standardized CVC bundles, rational antibiotic use, hand hygiene, and routine environmental cleaning/disinfection reduce colonization pressure and limit the spread of less-susceptible species. These measures complement—rather than replace—timely antifungal therapy and help prevent outbreaks and unnecessary broad-spectrum exposure [5,11,12,17,19,21,22,43,45,57,58].

### 2.4. Risk Factors and Therapeutic Decisions

In newborns—and especially critically in preterm infants with VLBW/ELBW—the immaturity of the innate and adaptive immune systems, together with the fragility of the mucocutaneous barriers and early colonization of the gastrointestinal tract, considerably increases vulnerability to candidemia and IC. The presence of invasive devices such as CVCs, endotracheal tubes, and catheters facilitates the breakdown of these defensive barriers and provides favorable surfaces for biofilm formation. The use of broad-spectrum antibiotics—particularly third-generation cephalosporins and carbapenems—promotes dysbiosis and favors the selection of *Candida* spp. Additional factors such as severe respiratory disease requiring MV, prolonged use of TPN, and the need for central vascular access cumulatively increase the risk. Likewise, procedures such as gastrointestinal surgery or events such as necrotizing enterocolitis facilitate the translocation of *Candida* spp. into the bloodstream. This set of determinants has been consistently reported in systematic reviews and clinical cohorts, although with variability depending on the healthcare context and local support and prevention practices [2,3,4,5,8,12,39,43,45,46,47].

In Latin America, neonatal candidemia maintains a dual pattern, with *C. albicans* and *C. parapsilosis sensu lato* (s.I.) predominating as etiological agents, closely linked to the use of invasive devices and intensive clinical support. At the same time, the emergence of non-*albicans* species with more variable antifungal profiles has been observed, adding to the growing challenge posed by *C. auris*. Among the most frequently associated clinical determinants are prematurity, VLBW, the presence of CVC, the use of TPN, MV, and previous exposure to antibiotics. These factors form the basis for adequate risk stratification and the design of specific prevention packages for the neonatal setting [2,4,5,9,12,14,15,16,17,18,22,23,24,25,26,39,43,45,47,49,51,55]. From a therapeutic perspective, regional experience supports the use of d-AmB or FCZ in well-defined contexts. However, the heterogeneity of antifungal susceptibility patterns and the circulation of *C. auris* require early identification at the species level, systematic AFST, and a reassessment of early access to echinocandins in high-risk NICUs [2,3,4,5,9,10,11,12,13,15,19,21,40,41,46]. Reducing the mortality associated with neonatal candidemia requires a comprehensive approach that includes early diagnosis, immediate initiation of active antifungal treatment, timely removal or replacement of invasive devices, and standardization of essential practices: hand hygiene, safe TPN management, CVC care packages, and targeted environmental disinfection [1,3,4,5,6,8,12,22,43,45,47,57]. Likewise, it is critical to implement neonatal surveillance networks that integrate comparable antifungal panels, particularly for echinocandins, and reports by species, as support for clinical decision-making and the adaptation of empirical treatment to the particular conditions of each Latin American NICU [5,6,9,10,12,13,14,15,16,17,19,25,28,49,51,52]. Table 2 summarizes the distribution of species and prevalent risk factors in these units.

Risk factors can be grouped into four interrelated areas:

1. *Host:* prematurity—especially in VLBW/ELBW neonates—markedly increases susceptibility to candidemia and IC due to immature skin and mucosal barriers, an underdeveloped innate immune response, and early gastrointestinal colonization by *Candida* spp. In NICUs with a high infection burden, FCZ prophylaxis may be considered for selected high-risk subgroups under strict criteria and with ongoing monitoring for toxicity and potential resistance [2,4,43,45,46,47].
2. *Devices and nutrition:* CVCs and TPN are key risk factors, largely through facilitation of biofilm formation. Mechanical ventilation and other invasive accesses further increase exposure time, provide additional portals of entry, and are associated with longer hospitalization and cumulative antimicrobial exposure [2,4,5,6,9,15,25,39,43,45,47].
3. *Antimicrobial pressure and adjuvant drugs:* broad-spectrum antibiotics disrupt the commensal microbiota and promote *Candida* overgrowth and colonization. Corticosteroids and H_2_ blockers may further increase risk, and gaps in IPC practices can sustain transmission and environmental persistence within NICUs [2,3,4,5,6,12,43,45,47].
4. *Unit ecology and emerging pathogens:* in Latin America, *C. parapsilosis sensu lato* (s.I.) is frequently predominant in neonatal candidemia and is closely linked to invasive device use, with documented contact transmission and outbreaks associated with lapses in CVC care bundles, TPN handling, and hand hygiene [5,9,12,15,19,39,57]. In Colombia and other countries in the region, the emergence of *C. auris* adds risk because of its capacity for healthcare transmission and multidrug resistance; neonatal outbreaks highlight the role of prior colonization, invasive devices, and IPC failures in progression to invasive infection, supporting the need for prompt isolation, contact screening when feasible, and timely therapy optimization guided by identification and AFST [20,21,27,28,30,31,32,33,34,37,56]. The concept of “colonization pressure”, including concurrent colonization of infants and healthcare personnel, remains an important precursor to invasive episodes [5,11,28,55,56].

Within this framework, we focus on the key determinants that inform risk stratification and provide the basis for meaningful regional comparisons, while reserving a more detailed discussion of bedside prevention and surveillance for later [2,4,5,8,12,39,43,45,47,48].

### 2.5. Mortality, Outcomes, and Modifiable Factors

Neonatal candidiasis continues to be associated with significant mortality and an elevated risk of neurodevelopmental sequelae, especially in newborns with VLBW or ELBW. In these patients, the immaturity of the immune system, frequent exposure to invasive devices, and multiple colonization—skin, mucous membranes, and gastrointestinal tract—contribute to a more unfavorable prognosis. Among the factors most consistently associated with adverse outcomes are: VLBW or very premature gestational age; clinical severity at diagnosis (e.g., shock, need for ventilatory or inotropic support); prolonged presence of CVC; delay in initiating effective antifungal treatment; and, finally, the local microbiological context—species isolated and antifungal resistance patterns prevalent in that NICU or region—[1,2,3,4,5,6,8,12,43,45,46].

Clinical practice guidelines agree on the critical importance of “time to treatment”: promptly initiating an active antifungal agent and ensuring control of the infectious focus—through removal or replacement of the CVC and drainage of collections when appropriate—is associated with lower mortality and better clinical prognosis in neonates with candidemia or IC [1,2,3,4,12,43,45,46,47]. In ELBW newborns, in addition to the high risk of acute mortality, persistent sequelae have been frequently documented: neurodevelopmental disorders, sensory deficits (auditory or visual), and long-term complications, which considerably increase the burden of disease beyond hospital discharge [1,6,43,45].

For reference, at HIC, the EUROCANDY study—conducted in 10 European countries between 2005 and 2015—reported a 30-day mortality rate of 14.4% in the overall pediatric cohort and 18.3% in NICU patients [8]. In contrast, the neonatal substudy of the NeoOBS project, conducted in LMICs, described a 28-day mortality rate of close to 22%, with a predominance of *C. parapsilosis sensu lato* (s.I.) (≈30% of isolates) and *C. auris* (≈14%), as well as a high proportion of FCZ-resistant isolates [5,6]. This scenario was associated with less favorable outcomes, especially when the initial antifungal treatment was not adjusted in a timely manner according to the antifungal susceptibility profile [5,6].

In Latin America, hospitalized newborns face a more complex prognosis for candidemia compared to older children, as documented in multiple multicenter studies. This difference is largely explained by the high burden of risk factors characteristic of the neonatal environment, such as the presence of CVCs, TPN, and prolonged ventilatory support. In addition, a predominance of non-*albicans* species has been described, especially *C. parapsilosis sensu lato* (s.I.), whose transmission is often linked to failures in infection control practices within NICUs [9,12,13,19,25]. Recent research in Brazil has confirmed that prolonged CVC retention, extreme prematurity, and the need for intensive support are directly associated with higher neonatal mortality. Elevated MICs against echinocandins have also been reported in isolates of *C. parapsilosis sensu lato* (s.I.) and *N. glabratus*, posing an additional challenge for the treatment. In this context, accurately selecting the initial antifungal agent and adjusting the treatment regimen according to the species and its sensitivity profile becomes essential for improving clinical outcomes [9,10,11,14,15,17,55].

In Colombia, data from a pediatric intensive care unit (ICU) in a tertiary center—including the neonatal population—reported a mortality rate from candidemia of approximately 36%, where both delays in initiating appropriate antifungal therapy and markers of clinical severity upon admission were independently associated with adverse outcomes [20,40]. The emergence of *C. auris* in NICUs—evidenced during a neonatal outbreak affecting multiple newborns—introduces an additional risk, given its multidrug resistance and ability to persist in the hospital environment. In such scenarios, the prognosis depends critically on early detection, contact isolation, effective environmental cleaning, and the selection of an active antifungal guided by the MIC and the identified circulating clade [21,28,30,31,32,33,34,35,37,38]. Table 2 summarizes, by country and setting, incidence (according to denominator: LB or admissions) and mortality (D28/D30 or hospital), showing a downward trend in HICs and heterogeneously higher rates in LMICs and several Latin American countries.

There are important differences between species that have direct implications for the clinical management of neonatal candidemia. In NICU neonates, *C. parapsilosis sensu lato* (s.I.) is a key pathogen: although its mortality may be comparable—or even slightly lower—than that of *C. albicans* when CVC control is achieved, and a targeted treatment is initiated, its marked ability to form biofilms and documented resistance to FCZ in certain contexts require an aggressive strategy. This strategy should include careful management of CVC and appropriate antifungal selection aimed at preserving this potential prognostic benefit [5,9,10,11,15,19,44,55]. On the other hand, *C. auris* is associated with high mortality and significant persistence in the hospital environment; in settings where there are no robust outbreak control protocols or active and early antifungal treatment, the risk of adverse outcomes increases significantly [21,28,30,31,32,34,35,37].

Practical implications for reducing mortality in the NICU:

1. *Early and appropriate antifungal treatment:* in high-risk newborns with strong clinical suspicion of candidemia or IC, effective antifungal treatment should be initiated without delay. Initial choice should be guided by the most likely species and, when available, AFST results, aiming to minimize delays—ideally initiating therapy within 24–48 h of clinical suspicion [2,3,4,5,12,43,45,46,47].
2. *Control of the infectious focus:* CVC removal or replacement should be pursued as early as feasible. Other potential foci should be actively assessed and managed (e.g., drainage of collections when indicated), and follow-up blood cultures should be obtained until clearance is documented. Treatment duration should be individualized based on source control and clinical response [2,3,4,12,46].
3. *Strategy based on the predominant species and local epidemiology:* in NICUs with high prevalence of FCZ-resistant *C. parapsilosis sensu lato* (s.I.) or documented circulation of *C. auris,* empiric azole therapy should be avoided. Instead, d-AmB or an echinocandin should be considered, with dosing tailored to gestational age and neonatal pharmacokinetics. Planned de-escalation should rely on favorable AFST results and clinical stability [2,3,4,5,11,12,15,19,28,40,41,46].
4. *Prevention of sequelae and reduction of long-term risk:* reducing unnecessary device exposure, limiting prolonged TPN and broad-spectrum antibiotics, and strengthening IPC practices are key to prevention. In VLBW/ELBW survivors, structured neurodevelopmental follow-up is also important to detect sequelae early and mitigate longer-term morbidity associated with neonatal fungal sepsis [2,12,39,43,45,47].

## 3. Discussion and Global Perspectives: Regional Comparison, Emerging Resistance, and Lines of Action

In Latin America, the available information on candidemia and other invasive fungal infections (IFIs) in newborns remains limited, fragmented, and marked by considerable methodological heterogeneity. Many studies in the region have focused on pediatric populations or immunocompromised patients outside the neonatal period—for example, children with cancer, transplants, congenital immunodeficiencies, or HIV. In contrast, studies specifically targeting newborns are less frequent, tend to be single-center, and have small sample sizes [9,12,14,15,16,19,20,23,24,25,26]. This lack of data highlights a significant gap in knowledge compared to that generated by HICs and international collaborative initiatives, where candidemia and neonatal IC are recognized as significant causes of LOS, with high morbidity and mortality rates and high resource consumption in NICUs [1,3,4,5,6,7,8,18,43,45]. A recent meta-analysis conducted in LMICs estimated an overall incidence of neonatal IC of 2.6% (95% CI: 2.2–3.0), with a significant proportion of FCZ-resistant isolates, and a combined case fatality rate of approximately 18.7% (95% CI: 15.5–22.1)—data that underscore the remarkable burden of disease in these settings [6].

In this context, newborns—especially those with VLBW—are the most vulnerable group to candidemia. This high susceptibility is associated with several widely recognized clinical factors: the need for MV, prolonged use of broad-spectrum antibiotics, parenteral nutrition (usually in the context of prolonged fasting, such as after gastrointestinal surgery or following an episode of necrotizing enterocolitis), and the sustained use of CVCs. To these determinants is added the immunological immaturity inherent in the early neonatal period [1,5,6,24,43,45,47]. The data available in Latin America reproduce this same risk pattern: most cases of candidemia are concentrated in highly complex neonatal care units and in premature newborns with VLBW. However, there are still significant variations in the way risk factors are defined and in the criteria used to calculate incidence, making direct comparisons difficult between studies [14,15,16,24,25,26].

In most studies, *C. albicans* continues to be the most frequently isolated species in cases of neonatal candidemia. However, a progressive shift in etiological distribution toward non-*albicans* species has been documented, with *C. parapsilosis sensu lato* (s.I.) standing out as the most prevalent in multiple NICUs in Latin America, along with an increase in the detection of *C. tropicalis* and *M. guilliermondii* [5,6,9,18,19,23,24,26,41,42,44]. This transition has important clinical and epidemiological implications, as species such as *C. parapsilosis sensu lato* (s.I.) have a remarkable ability to form biofilms on intravascular devices, facilitating nosocomial outbreaks and complicating treatment [9,18,41,44,53]. At the same time, a decrease in sensitivity to antifungals widely used in neonatology, such as d-AmB and FCZ, has been observed. Neonatal isolates resistant to FCZ have been reported in Brazil—particularly in the state of Pernambuco—and in other Latin American cohorts. In the LMIC meta-analysis, approximately 25% of clinical isolates exhibited antifungal resistance [6,9,10,11,15,19]. Although these data should not be generalized without careful contextual analysis, they underscore the urgency of implementing continuous antifungal susceptibility monitoring in the region.

In Latin America, NICUs have already faced outbreaks caused by *C. auris*, an emerging yeast known for its multidrug resistance and marked ability to spread in hospital settings. This species has been responsible for both neonatal and pediatric outbreaks in various countries in the region, including Colombia [21,27,28,29,30,32,35,36,37,38,59]. Alongside its expansion in the United States and Europe, *C. auris* has become established as a high-risk pathogen for public health, which has led to its inclusion as a priority in international lists of critical fungi [31,32,33,34,35,38]. Faced with this threat, NICUs are forced to rigorously implement a series of operational measures: immediate isolation of patients and their contacts according to the level of risk, strict compliance with hand hygiene protocols, maximum care in performing invasive procedures, use of disinfectants effective against *C. auris*, and structured environmental cleaning protocols [28,29,30,34,35,36,37,38]. The effectiveness of these interventions depends crucially on accurate species identification and reliable antifungal susceptibility testing; therefore, the use of robust diagnostic platforms that minimize errors in the detection of emerging yeasts is recommended [9,11,29,30,31,35,37].

In general—although figures vary between studies—there is clinical consensus: both candidemia and IC in neonates are serious infections that are complex to manage and have a very significant impact on survival and quality of life, even when in vitro studies show favorable antifungal sensitivity [1,3,4,5,6,7,8,18,43,45]. The prognosis depends largely on a combination of patient-specific factors—such as extreme prematurity, VLBW, or comorbidities such as necrotizing enterocolitis or bronchopulmonary dysplasia—along with variables related to the healthcare environment: frequent exposure to invasive procedures, delays in obtaining blood cultures, delays in initiating appropriate antifungal treatment, and difficulties in removing catheters in a timely manner. This pattern has been reported in Latin American cohorts as well as series from the US, Europe and Asia [5,6,7,8,14,15,16,23,24,25,26,43,45]. An additional point of concern is the lack of consistent data on long-term consequences: neurodevelopmental, sensory, or motor impairments among survivors. Unlike reports in HICs, where this risk has been documented, in Latin America these effects continue to be understudied and underreported [1,6,43,45]. This is largely due to the absence of structured follow-up programs after hospital discharge, which limits our understanding and comprehensive approach to the long-term consequences of neonatal IC.

In many of the series reviewed, the source of the infection is rarely thoroughly investigated. Although reasonable hypotheses have been put forward—such as inadequate hand hygiene, perinatal mucosal skin colonization, cross-transmission by healthcare personnel, or intestinal translocation in contexts of inflammation or after prolonged parenteral nutrition—few studies systematically integrate data on colonization, molecular typing, and clinical correlation in newborns [5,6,25,43,44,55,56]. Some experiences in Latin American NICUs document considerable rates of yeast colonization in both neonates and healthcare personnel, reinforcing the idea of a nosocomial reservoir as a possible origin of many episodes of candidemia [55,56]. In an isolated case, a study in Mexico observed an increase in the incidence of candidemia during the warm months of the year [16], suggesting a possible environmental or seasonal influence. However, these associations should be interpreted with caution and require validation through surveillance systems that incorporate climatic variables. This aspect is particularly relevant given the growing body of evidence linking climate change to the emergence of opportunistic mycoses, especially those caused by *C. auris*—an emerging yeast whose thermotolerance and environmental adaptation may have been facilitated by global warming [6,31,60,61,62].

**Priority lines of action for NICUs in Latin America.**

Taken together, these challenges point to several priorities for NICUs across Latin America. Although the way these measures are implemented will vary according to local resources and infrastructure, they are central to improving surveillance, informing clinical decisions, and strengthening prevention and follow-up across the region:

1. *Standardization of epidemiological indicators:* there is a clear need to harmonize the definitions and denominators used to report candidemia and related infections, including live births, episodes of LOS, microbiologically confirmed BSI, and HAIs. Without greater consistency in reporting, comparisons across institutions remain difficult, and the burden of disease in LMICs is likely to be underestimated or incompletely captured [5,6,23,24,26,57].
2. *Improving mycological diagnosis and antifungal surveillance:* whenever possible, neonatal *Candida* isolates should be identified to the species level and tested routinely for antifungal susceptibility. Where resources allow, diagnostic strategies should also include methods that reliably detect *C. auris*, with results interpreted according to CLSI- or EUCAST-aligned ECOFF/ECV frameworks. Expanding this capacity is important not only for early recognition of resistance patterns, but also for building stronger regional surveillance systems [2,6,9,11,29,30,31,34,37,38].
3. *Optimization of antifungal treatment:* both empiric and targeted antifungal therapy should be guided by local species distribution and susceptibility patterns, and empiric regimens for high-risk neonatal LOS should be reviewed periodically. Current guidance consistently supports early appropriate treatment and timely CVC management, particularly in infections involving biofilm-associated species or persistent candidemia. At the same time, these recommendations need to be applied in light of local realities, including the availability of agents such as echinocandins and L-AmB and other unit-level constraints [1,2,3,4,12,43,45,47,48].
4. *Strengthening infection prevention and control (IPC):* particular emphasis should be placed on strict hand hygiene, standardized catheter-care bundles, safe preparation and administration of TPN and medications, and routine audit and feedback. Experience from outbreaks involving *C. parapsilosis sensu lato* (s.I.) and *C. auris* shows that sustained adherence to these measures can reduce transmission and lower the incidence of candidemia [17,18,21,22,28,29,30,31,32,33,34,35,36,37,38,43,53,58,59].
5. *Building collaborative surveillance systems:* expanding multicenter active surveillance would allow more consistent reporting of species distribution, AFST profiles, and clinical outcomes, including mortality and sequelae. Regional initiatives and LMIC meta-analyses have already shown the value of coordinated surveillance for identifying gaps and informing policy. In this context, a Latin American NICU network could help reduce fragmentation and provide more robust regional estimates [5,6,9,10,22,57].
6. *Post-discharge follow-up of survivors:* survivors of candidemia or IC should undergo longitudinal follow-up using standardized assessments of neurodevelopment, hearing, vision, and longer-term functional outcomes. This need is supported by evidence from HICs and is increasingly relevant in the region, where concern about the sequelae of fungal sepsis in early life continues to grow [1,6,17,43,45,49,50,51,52,57].

In summary, Latin America faces a double challenge: on the one hand, the persistence of *C. albicans* as the predominant agent in many NICUs; on the other, the sustained increase in non-*albicans* species—particularly *C. parapsilosis sensu lato* (s.I.)—and the emergence of *C. auris* as a significant threat to the safety of newborns [5,6,9,11,14,15,16,18,19,20,23,24,25,26,27,28,30,40,43,44,45,58]. Overcoming this situation requires transforming scattered evidence into contextualized clinical recommendations, which involves standardizing methods, strengthening the capacities of microbiology laboratories, implementing programs for the rational use of antifungals, and designing preventive strategies adapted to the structural realities of each NICU [6,9,10,12,22,31,43,45,47,48]. Only with this comprehensive approach will it be possible to convert the accumulated knowledge on neonatal candidemia into concrete improvements in the survival and quality of life of the most vulnerable newborns.

### 3.1. Implications of Emerging Resistance (Especially C. auris)

*Candidozyma auris* has emerged as a clinically relevant cause of healthcare-associated infection in NICU settings, particularly in LMICs, where cases have been reported predominantly among preterm infants with VLBW exposed to CVCs, TPN, mechanical ventilation, and prolonged broad-spectrum antibiotics; outbreak-associated neonatal mortality approaching ~40% has been described [6,35,45]. A key concern is its frequent multidrug resistance, including high rates of reduced susceptibility to fluconazole and variable activity of amphotericin B, which reinforces the need for rapid, accurate species identification and routine AFST to guide therapy and infection-control decisions [5,11,21,27,30,35,38,43,45,46]. In Latin America, NICU outbreaks reported in countries such as Colombia and Venezuela highlight the importance of early activation of contact precautions, screening of contacts when feasible, rigorous environmental disinfection with agents effective against this organism, and prompt evaluation of removable invasive devices [11,21,30,34,35,37,38,43].

In the specific context of NICUs, the practical implications are clear and urgent. First, whenever *Candida* spp. is identified in blood cultures, cerebrospinal fluid, or other sterile sites, species-level identification and AFST should be performed systematically, without assuming azole or polyene susceptibility [5,11,21,27,30,35,38,43,45,46]. Second, in settings where *C. auris* is present or an outbreak has been confirmed, echinocandins should be considered for empiric therapy in high-risk neonatal candidemia, and subsequent treatment should be individualized based on the species and MIC profile, the clinical syndrome (including possible central nervous system involvement), and drug penetration into the CNS [5,11,21,35,38,45,48]. Finally, when *C. auris* infection is suspected or confirmed, institutional control protocols should be activated without delay, including contact precautions, strict hand hygiene, environmental disinfection with agents effective against this organism, contact screening when feasible, and early assessment and management of removable invasive devices such as CVCs and endotracheal tubes [11,21,30,34,35,37,38,43].

Finally, in NICUs, selective pressure from prolonged broad-spectrum antibiotic exposure and frequent invasive procedures may facilitate *Candida* overgrowth and the selection and transmission of multidrug-resistant strains, reinforcing the need for integrated antimicrobial and antifungal stewardship [5,6,17,43,45,49,50,57]. Practical priorities include standardized CVC care, prudent use and early de-escalation of antibiotics when appropriate, safe TPN practices, optimization of ventilatory support, and regular audit-and-feedback cycles, combined with active microbiological surveillance and strict IPC measures [5,11,22,38,43,48,57].

### 3.2. Environmental and Seasonal Considerations Relevant to NICU Epidemiology

Although neonatal candidemia is mainly determined by NICU-level factors—including prematurity, invasive device exposure, antimicrobial use, and adherence to infection prevention and control—the care environment becomes clinically relevant when it favors horizontal transmission. In this setting, the key environmental concerns are not outdoor ecological conditions, but rather features of the unit itself and the processes of care within it. These include persistence of yeasts on high-touch surfaces and shared equipment, contamination during medication or TPN preparation and handling, and wet-area reservoirs such as sinks and drains that may increase colonization pressure. Such factors are particularly important during clusters or outbreaks caused by biofilm-associated species such as *C. parapsilosis sensu lato* (s.I.) and *C. auris*, where environmental persistence and inadequate cleaning or disinfection may sustain transmission. Environmental considerations are therefore best understood here as part of actionable NICU epidemiology, with emphasis on cleaning targets, review of TPN and CVC workflows, and risk-based environmental sampling when outbreaks are suspected, rather than as a broader discussion of fungal ecology [60,61,62].

Seasonality has been reported intermittently in neonatal candidemia datasets, but the evidence remains limited and does not support a causal association. When seasonal peaks are identified, they are likely to reflect indirect influences, including changes in unit occupancy, staffing ratios, workflow disruptions, temperature or humidity effects on organism persistence, or variation in hand hygiene and cleaning performance during periods of greater demand. In this review, seasonality is therefore treated as an exploratory surveillance variable. Incorporating basic meteorological measures or calendar periods, such as warmer months, may be useful for hypothesis generation and for prompting targeted IPC review, but interpretation should remain centered on the core NICU determinants, including device utilization, antibiotic exposure, adherence to CVC and TPN bundles, and outbreak control measures [6,16,60,61,62].

### 3.3. Comparison with European Studies

The EUROCANDY multicenter retrospective study, conducted in 23 hospitals in ten European countries (Belgium, Denmark, Germany, Greece, Italy, the Netherlands, Norway, Serbia, Spain, and the UK), reported a total of 1395 episodes of pediatric candidemia. Of these, 422 cases (36.4%) corresponded to newborns admitted to the NICU [8]. In the neonatal cohort, the most common species was *C. albicans*—approximately 60% of isolates—followed by *C. parapsilosis sensu lato* (s.I.) with a prevalence of around 26–28%. Isolates of *C. tropicalis, N. glabratus*, and *P. kudriavzevii* were also recorded, in addition to a small number of mixed candidemias (~0.7%) in which multiple species were identified [8]. The 30-day mortality rate was 14.4% in the general pediatric population; however, in neonates hospitalized in the NICU—and in those with infections caused by less common species such as *P. kudriavzevii* or in polyfungal cases—this figure rose to around 18%. In contrast, infections caused by *C. albicans* and *C. parapsilosis sensu lato* (s.I.) showed lower mortality rates that were similar to each other [8]. A notable epidemiological finding was geographical variability: *C. albicans* predominated in northern and western European countries, while a higher proportion of *C. parapsilosis sensu lato* (s.I.) was observed in southern countries [7,8].

In Italy, a nine-year retrospective review in a university NICU reported 41 cases of neonatal candidemia, representing an approximate incidence of 3 episodes per 100 admissions [18]. In that series, the predominant isolated species was *C. parapsilosis sensu lato* (s.I.) sensu stricto, responsible for between 58.5% and 59% of cases, followed by *C. albicans* with 34.1% [18]. Other less common yeasts included *N. glabratus, M. guilliermondii*, and *C. orthopsilosis*, each with a frequency of approximately 2.4% [18]. The affected newborns were predominantly premature with VLBW, with a median gestational age of 30 weeks (interquartile range 29–31) and birth weight around 1100 g (interquartile range 900–1345 g) [18]. All episodes were classified as neonatal LOS. Recurrent risk factors included gestation < 32 weeks, VLBW, prolonged hospital stay (>7 days), use of CVC, MV, and prolonged exposure to broad-spectrum antibiotics [18]. A relevant finding was that neonates with non-*albicans* candidemia tended to have longer hospital stays and a more prolonged requirement for TPN compared to those infected with *C. albicans*, reinforcing the association between prolonged invasive support and the predominance of non-*albicans* species in the neonatal setting [18]. However, the study did not include a detailed analysis of antifungal sensitivity or provide mortality data broken down by species, limiting the interpretation of its impact on clinical outcomes and the assessment of resistance [18].

Overall, the patterns observed in European studies are sufficiently consistent with those reported in Latin America and other LMICs. In general, *C. albicans* predominates, with *C. parapsilosis sensu lato* (s.I.) playing a notable role, especially in NICUs in southern Europe. These epidemiological findings share risk factors with the Latin American series: prematurity, VLBW, CVC use, MV, TPN, and prolonged treatment with broad-spectrum antibiotics [5,6,7,8,9,18,23,24,26,43,44,45]. However, there are important differences: while the European multicenter study did not include antifungal susceptibility data, and the Italian series did not provide specific mortality figures by species, several Latin American studies and a meta-analysis in LMICs do describe neonatal mortalities due to candidemia ≥ 20% and a sustained increase in non-*albicans* species with reduced susceptibility to azoles and polyene antifungals [5,6,7,9,10,15,19,23,24,26]. In addition, the Italian series reported an association between prophylactic FCZ use in ELBW neonates and an increase in the incidence of non-*albicans* candidemia. Although this finding does not establish causality, it suggests possible selective pressure. This phenomenon—consistent with observations from other regions—highlights the need for strict surveillance of antifungal prophylaxis regimens and monitoring of the local *Candida* ecology in NICUs [1,5,7,8,12,18,43,45,47].

### 3.4. Recommendations for Surveillance and Prevention Policies

From a policy perspective, reducing neonatal candidemia in the NICU requires integrated surveillance and prevention strategies directed at the main modifiable drivers of invasive disease, particularly prolonged exposure to invasive devices, suboptimal CVC and TPN practices, and unnecessary use of broad-spectrum antibiotics. In high-risk settings, these measures may be complemented by risk-based antifungal prophylaxis, reserved for units with a clearly documented baseline burden or recurrent outbreaks and guided by ongoing surveillance of local species distribution and antifungal susceptibility patterns [5,6,9,22,43,45,47,48].

Within this framework, empirical antifungal treatment should be reserved for neonates with a high clinical and epidemiological risk profile. It has been suggested that empirical treatment be initiated in extremely premature infants (<27 weeks’ gestation) with LOS accompanied by thrombocytopenia, particularly in the presence of additional risk factors such as CVCs or peripherally inserted central lines, prolonged TPN with lipid emulsions, mechanical ventilation, recent exposure (≤7 days) to third-generation cephalosporins or carbapenems, multiple antibiotic courses, or corticosteroid use [43,45,47]. In practice, prophylaxis has most commonly involved FCZ—and to a lesser extent nystatin—whereas in many centers d-AmB remains the treatment of choice for confirmed fungemia, often reflecting local drug availability and cost considerations [2,3,12,43,45,46,47,48].

Accordingly, we highlight the determinants that most strongly shape risk stratification and allow meaningful regional comparisons, while presenting prevention and surveillance priorities in a streamlined, practice-oriented form [2,4,5,8,12,39,43,45,47,48].

Because species distribution and antifungal susceptibility patterns may vary substantially across hospitals and regions, surveillance objectives—including the detection of *C. auris*—and prevention priorities should be tailored to local epidemiology [5,6,7,9,10,11,19,23,24,26,43,45]. Based on the available evidence, the following strategies may help strengthen active surveillance and prevention of invasive fungal diseases, particularly candidemia, in NICU settings:

1. Epidemiological and microbiological surveillance
  Active surveillance in the NICU should incorporate standardized denominators (e.g., per 1000 admissions or catheter-days), species-level identification, and routine AFST, together with reliable detection of *C. auris* and prompt communication between the laboratory and the clinical team. During clusters or outbreaks, targeted colonization screening and risk-based environmental sampling may help clarify transmission routes and support containment, according to local protocols [5,6,9,10,22,43,45]. Screening should focus primarily on neonates and, where supported by local IPC policies, selected contacts; routine screening of healthcare personnel is generally not warranted unless indicated by outbreak investigations [5,43,48,55].
2. Hand hygiene
  High levels of hand-hygiene adherence should be sustained through regular audit and feedback, point-of-care reminders, and staffing or workflow measures that help maintain compliance during periods of high occupancy [11,22,34,35,45].
3. Environmental cleaning and disinfection
  Cleaning and disinfection protocols should include agents with documented activity against *Candida* spp. and *C. auris*, with standardized attention to high-touch surfaces, shared equipment, and wet reservoirs such as sinks and drains. During outbreaks, these measures should be reinforced by audit and feedback and by terminal cleaning, while avoiding exclusive reliance on quaternary ammonium compounds in settings where reduced activity has been reported [11,34,35,38,45].
4. Prevention of CVC-related infections
  Consistent implementation of standardized protocols for CVC insertion and maintenance—including sterile technique, careful site selection, scheduled dressing care, and daily review of device necessity—has been associated with substantial reductions in bloodstream infections in NICUs and pediatric units [5,22,43,57,58].
5. Rational use of antibiotics and antifungals
  Antimicrobial and antifungal stewardship should aim to reduce unnecessary exposure to broad-spectrum antibiotics, encourage early reassessment of empiric therapy, and support periodic review of indications for empirical treatment and prophylaxis in light of local species distribution and AFST results [5,11,22,38,43,45,48,57].
6. Contact precautions and cohorting
  When *C. auris* or other azole-resistant yeasts are suspected or identified, contact precautions should be implemented promptly. Isolation or cohorting, when feasible, should be accompanied by reinforcement of PPE use and hand hygiene, together with coordinated screening and enhanced cleaning in collaboration with IPC and microbiology teams [11,21,30,34,35,37,38,43,45].
7. Additional non-pharmacological strategies
  Non-pharmacological interventions should not be presented as specific strategies for candidemia prevention. Kangaroo care should continue to follow standard neonatal indications; probiotics are not recommended solely for prevention of IFD and may carry a risk of fungemia in highly vulnerable infants; and G-CSF is not recommended for prevention of IC [45,48,58].

Overall, preventing neonatal candidemia—especially that caused by resistant species like *C. auris*—in Latin America requires a comprehensive approach: active surveillance, systematic implementation of preventive measures, rational use of antimicrobials, and rigorous infection control practices. Only in this way will it be possible to reduce the burden of disease in the region’s NICUs [5,6,9,11,22,43,45,47,48].

## 4. Conclusions

Invasive *Candida* spp. infections remain a major cause of LOS, mortality, and neurological sequelae in neonates—with a particularly severe impact on newborns with VLBW or ELBW. The combination of factors such as immune system immaturity, incomplete mucocutaneous and mucosal barriers, early colonization, and the frequent need for invasive interventions makes NICUs especially vulnerable to these infections. In Latin America, neonatal candidemia reveals a dual pattern: while *C. albicans* continues to be a predominant species, there is a sustained increase in non-*albicans* species—especially *C. parapsilosis sensu lato* (s.I.)—closely associated with the use of medical devices, biofilm formation, and horizontal transmission between patients or through the environment. Added to this panorama is the emergence and spread of C. auris, a multidrug-resistant fungus that is difficult to identify, responsible for NICU outbreaks and associated with alarming neonatal mortality. In a context of limited diagnostic capacity and variable adherence to preventive measures, this dynamic further intensifies the heterogeneity in incidence and clinical outcomes across the region. This dynamic, in a context of limited diagnosis and variability in adherence to preventive measures, intensifies the heterogeneity in incidence and clinical outcomes in the region.

From the therapeutic point of view, “time to treatment” emerges as a key modifiable factor: promptly initiating an active antifungal in suspected cases, accompanied by effective control of the infectious focus—including timely catheter removal or exchange and drainage of collections when present—improves the chances of survival. Empiric therapy selection should be guided by local ecology and known resistance patterns. In NICUs with a high prevalence of resistant *C. parapsilosis sensu lato* (s.I.) or circulation of *C. auris*, azoles may be unreliable, favoring d-AmB or echinocandins, with subsequent de-escalation guided by species identification and MICs.

NICUs in Latin America share a common risk profile: extreme prematurity, VLBW, frequent use of CVC, TPN, MV, and prolonged administration of broad-spectrum antibiotics. These factors should serve as criteria for risk stratification to clearly define which subgroups could benefit from antifungal prophylaxis and to guide preventive interventions adapted to the regional context. Structured interventions—such as best practice packages for CVC and TPN management, optimization of antibiotic use, systematic hand hygiene, and targeted environmental cleaning—are essential to reduce selection pressure and limit the transmission of less susceptible species, without relying exclusively on pharmacological treatment.

However, the regional evidence remains limited, fragmented, and heterogeneous: there are variations in operational definitions, denominators used, and methodologies for AFST. This gap highlights the urgent need to strengthen neonatal surveillance networks that integrate species identification, antifungal sensitivity profiles, and standardized clinical and laboratory data. Only in this way will it be possible to compare results between centers and countries. Similarly, studies that systematically assess the real impact of combined strategies—encompassing prevention, rational use of antimicrobials, tailored therapeutic profile, and access to new antifungal agents—on neonatal mortality and sequelae in survivors are urgently needed.

In summary, moving towards an effective response to neonatal candidemia in the region involves overcoming dependence on scattered data and adopting contextualized, empirical policies. This requires supporting three fundamental pillars: active surveillance by species and antifungal susceptibility, timely treatment based on local epidemiology, and robust prevention and “antimicrobial stewardship” programs adapted to the resources and specific characteristics of Latin American NICUs. Only in this way will it be possible to sustainably reduce both the mortality and disability associated with these infections in the neonatal population.

## Figures and Tables

**Table 1:** Neonatal Candidemia—Global and Regional Comparison. Studies included in Table 1 were identified using the narrative search strategy and eligibility criteria described in the Methods/Literature Search section (2008–2025; Latin American NICU/neonatal extractable data).

Region/Setting	Study (Design; Period)	Reported Incidence/Prevalence *	Key Species	Antifungal Resistance (Summary)	Comments/Findings	Refs.
Global—HICs (U.S.)	CDC population-based surveillance (4 areas), 2009–2015	Neonates: 31.5 → 10.7–11.8/100,000 LB (2009 → 2012–2015)	Mixed; *C. albicans* and non-*albicans* species predominate depending on age	Not designed for detailed AFST; useful for burden and outcome trends	Regulatory framework for stratification, prophylaxis, and de-escalation; standard on time to treatment initiation	[7]
Global—LMICs (NeoOBS)	Prospective cohort (14 hospitals/8 countries), 2018–2021	Population data not reported; NICU cohort	*C. albicans* ≈ 35%, *C. parapsilosis sensu lato* (s.I.) ≈ 30%, *C. auris* ≈ 14% (site variation)	High FCZ resistance in several centers; 0% MCF resistance in the pooled site report †	Mixed species: *C. albicans* ~35%, *C. parapsilosis sensu lato* (s.I.) ~30%, *C. auris* ~14%; significant FCZ resistance; 0% MCF resistance in the pooled data	[5]
Europe (EUROCANDY)	Multicenter retrospective, 10 countries, 2005–2015	NR (etiology and outcomes study)	*C. albicans* 52.5%, *C. parapsilosis sensu lato* (s.I.) 28% (in neonates, *C. albicans* 60.2%)	Not focused on AFST; guidance on distribution and mortality	HIC comparator for burden, species, and outcomes versus LMICs and LA	[8]
Latin America (7 countries)	Multicenter laboratory-based survey (PLOS One), 2008–2010	Global: 1.18/1000 admissions. Country: ARG 1.95; BRA 1.38; CHL 0.33; COL 1.96 *; ECU 0.90; HND 0.90; VEN 1.72	*C. albicans* 37.6%, *C. parapsilosis sensu lato* (s.I.) 26.5%, *C. tropicalis* 17.6%	Low overall resistance to azoles and echinocandins during the period	Greater prevalence of non-*albicans* species in children; *C. parapsilosis sensu lato* (s.I.) relevant in the neonatal subgroup	[19]
Brazil	NICU (3 hospitals), 2010–2014; publ. 2023	Candidemia prevalence 10.97% among neonates with suspected sepsis	*C. parapsilosis sensu lato* (s.I.) and *C. albicans* complex; non-*albicans* species ≈ 68%	Elevated MICs to echinocandins in *C. parapsilosis sensu lato* (s.I.)/*N. glabratus*; *C. haemulonii* complex with high MICs to AmB and FCZ		[15]
Chile (multicenter)	National multicenter prospective study, 2013–2017	Hospital incidence reported in text (not standardized to LB)	Regional pattern with high pediatric burden of *C. parapsilosis sensu lato* (s.I.)	Susceptibility profile consistent with other Latin American series	National network useful for comparison with other LA countries and with HIC	[13]
Mexico	NICU Guadalajara, 2015–2019	2.27/1000 LB	*C. albicans* 35.3%, *C. parapsilosis sensu lato* (s.I.) 30.6%, *N. glabratus* 31.8%; isolated cases of *C. lipolytica*	Local susceptibility profile; FCZ prophylaxis protective against IC	Reinforces the impact of devices and antibiotics; supports prevention measures and ASP/AFP programs	[16]
Argentina	LA sub analysis (2008–2010) + historical NICU series	1.95/1000 admissions (range 0.36–2.98)	*C. albicans* and *C. parapsilosis sensu lato* (s.I.) predominate in NICU	Low resistance during the evaluated period	Predominance of *C. albicans* ~36% and *C. parapsilosis sensu lato* (s.I.) ~36%; highlights the role of IPC	[19,42]
Peru (Lima–Callao)	Multicenter hospital-based, 2013–2015 (*Candida* BSI)	Global: 2.04/1000 admissions; by center 1.01–2.63	*C. albicans* 27.8%, *C. parapsilosis sensu lato* (s.I.) 25.3%, *C. tropicalis* 24.7%, *N. glabratus* 9.5%	FCZ resistance ≈ 2.5%; ANF and AmB 100% susceptible	Expands the Andean context; guides empirical selection and resistance surveillance	[23]
Panama (NICU)	NICU series (Rev Chil Pediatr), 2014–2016	141 infection episodes in the NICU during the period (no LB rate)	*C. parapsilosis sensu lato* (s.I.) 49%	No detailed AFST; emphasis on risk factors	*C. parapsilosis sensu lato* (s.I.) ~49%; reinforces timely device removal and strict IPC	[26]
Colombia	Descriptive series (pediatric *C. auris* + neonatal NICU outbreak), 2016–2017	Series/outbreaks (non-population-based)	Emerging *C. auris*; also *C. albicans* and *C. parapsilosis sensu lato* (s.I.)	Multidrug resistance in *C. auris* (azoles ± AmB); need for specific AFST	Requires specific AFST and containment strategies in the NICUs Example of neonatal outbreak; illustrates the urgency of species-level surveillance and control measures	[21,28]
Costa Rica (NICU)	NICU series, 1994–1998	NICU series (no population rate)	Predominance of *C. albicans*; non-*albicans* species present	No detailed AFST (pre-echinocandin era)	Classic study that established the neonatal risk model and the importance of prevention	[24]
Ecuador	LA sub analysis (2008–2010)	0.90/1000 admissions (range 0.39–1.39)	Higher proportion of *C. albicans* compared to other LA countries	Low global resistance in the survey		[19]
Honduras	LA sub analysis (2008–2010)	0.90/1000 admissions (range 0.38–2.41)	*M. guilliermondii* locally significant	Low global resistance in the survey		[19]
Venezuela	LA sub analysis (2008–2010) + historical neonatal series	1.72/1000 admissions (range 0.64–2.98)	*C. parapsilosis sensu lato* (s.I.) and *C. albicans* predominate	Variability between series in species and susceptibility		[19]

Original denominators are preserved (per 100,000 LB or per 1000 admissions). NR is indicated when the study does not report a standardized rate. Country-specific incidences from the Latin American laboratory-based study come from the multicenter PLoS ONE (values per 1000 admissions: Argentina 1.95; Brazil 1.38; Chile 0.33; Colombia 1.96 *; Ecuador 0.90; Honduras 0.90; Venezuela 1.72; global 1.18). As an international reference, CDC surveillance in the USA showed a decrease in neonatal incidence from 31.5 in 2009 to 10.7–11.8 per 100,000 LB in 2012–2015. In LMICs, NeoOBS reported D28 mortality ≈22% and a mixed species distribution with significant FCZ resistance, with no MCF-resistant isolates in the pooled data. * Colombia: incidence per 1000 admissions calculated in a single hospital; 0.69 (0.33–1.86) per 1000 patient-days. † NeoOBS reports high FCZ resistance in several sites and no MCF resistance in the analyzed set of centers. LB: live birth; HIC: high-income countries; LMICs: low- and middle-income countries; NICU: neonatal intensive care unit; CVC: central venous catheter; TPN: total parenteral nutrition; MV: mechanical ventilation; AFST: antifungal susceptibility testing; AmB: amphotericin B (deoxycholate or lipid formulations depending on study); FCZ: fluconazole; MCF: micafungin; MIC: minimum inhibitory concentration; D28/D30: mortality at day 28/day 30 from the index episode; IPC: infection prevention and control; LA: Latin America; BSI: bloodstream infection.

**Table 2:** Risk Factors and Mortality in Neonatal Candidemia.

Country/Setting	Study (Design; Period)	Neonatal Inclusion	Main Risk Factors	Mortality/Outcomes	Comments/Findings	Ref.
Global (guide)	IDSA Candidiasis Guideline (CPC; 2016)	N/A (regulatory framework)	ELBW/VLBW, CVC, TPN, broad-spectrum antibiotics, MV, multicolonization	Significant mortality in high-risk neonates; early initiation of active antifungal and source control reduce mortality; risk of neurodevelopmental impairment in ELBW	Reference in HIC for trends and impact of preventive measures post-2010	[2]
LMICs (multi-country)	NeoOBS (prospective; 2018–2021)	LMIC neonatal cohort	Low GA/BW, clinical severity, CVC, antibiotics; mixed by species	D28 ≈ 22%	D28 mortality ≈ 22%; LMIC context compared to HIC	[5]
Europe (HICs)	EUROCANDY (multinational retrospective ; 2005–2015)	Neonates and children; NICU sub-analysis	Prematurity/ELBW, ICU stay, devices, antibiotics	Pediatric 30-day mortality ≈ 14.4%; in NICU ≈ 18.3%	30-day mortality 14.4% pediatric global; 18.3% in NICU; HIC comparator	[8]
Latin America (multicenter pediatric)	Active pediatric candidemia surveillance; 23 hospitals/8 countries (prospective; 2008–2010)	Pediatrics with ≈29% neonates	Prematurity, NICU stay, CVC, TPN, MV	NR	High pediatric proportion; *C. parapsilosis sensu lato* (s.I.) common in children/neonates; marked variability between countries	[25]
Brazil (3 NICUs)	Cohort with AFST (2010–2014; publ. 2023)	Neonates in NICU	CVC, VLBW/ELBW, TPN, MV	Mortality associated with CVC and clinical severity; rate detailed in the original text	CVC associated with mortality; need for close monitoring of MIC and species	[15]
Chile	National multicenter (prospective; 2013–2017)	Includes neonates and pediatric population	CVC, broad-spectrum antibiotics, NICU stay	Hospital/30-day mortality reported in the study	National reference point for LA, useful as an intra-regional comparator	[13]
Mexico	NICU (incidence and factors; 2015–2019)	Neonates in NICU	Prematurity, CVC, antibiotics; FCZ prophylaxis with protective effect in VLBW	NR	Antibiotics, CVC, TPN, and MV associated with higher risk; prophylactic FCZ with protective effect adjusted for weight	[16]
Argentina (Northeast)	NICU (series; 2004)	Neonates in NICU	Prematurity, catheters, antibiotics	NR	Classic neonatal pattern; central role of CVC and IPC measures	[42]
Peru (Lima–Callao)	Multicenter hospital study of *Candida* BSI (2009–2011)	Includes pediatric and neonatal subgroups	Devices, antibiotics, severity at admission	Reported in-hospital mortality	30-day survival ≈ 60.4%; improves with early treatment	[23]
Panama	NICU (cases and controls; 2014–2016)	Neonates in NICU	Length of stay > 7 days, umbilical lines, surgery, meropenem	High lethality in the cohort	Higher lethality associated with *C. parapsilosis sensu lato* (s.I.); risks: length of stay > 7 days, umbilical lines, surgery, meropenem	[26]
Colombia (pediatric BSI)	Series of *C. auris* BSI in pediatrics (2014–2017)	Pediatrics including neonates	Devices, antibiotics, NICU stay; colonization/environment	High mortality; worse outcomes with multidrug-resistant profiles	NICU outbreaks and nosocomial transmission; importance of environmental control and IPC	[21]
Colombia (NICU outbreak)	*C. auris* outbreak in NICU (2016–2017)	Neonates in NICU	Environmental and contact transmission; device use	Significant lethality in the series; impact of contact isolation and targeted cleaning		[28]
Costa Rica (historical)	NICU (cohort; 1994–1998)	Neonates in NICU	Prematurity, CVC, TPN, antibiotics	Mortality ≈ 34%	Historical series illustrating the classic neonatal risk model	[24]

Note: The denominators of mortality reported by each study are preserved (e.g., 30-day mortality, in-hospital mortality, D28). When the source does not specify the denominator or does not include neonates separately, NR is indicated. This table complements Table 1 by focusing on the main risk factors, mortality or clinical outcomes, and the neonatal population included in the studies, comparing local and international settings. LB: live birth; ELBW: extremely low birth weight (<1000 g); VLBW: very low birth weight (<1500 g); GA/BW: gestational age/birth weight; CVC: central venous catheter; TPN: total parenteral nutrition; MV: mechanical ventilation; NICU: neonatal intensive care unit; ICU: intensive care unit; IPC: infection prevention and control; ASP: antimicrobial stewardship program; AFP: antifungal stewardship program; AFST: antifungal susceptibility testing; ECN: echinocandins; FCZ: fluconazole; AmB: amphotericin B; MCF: micafungin; MIC: minimum inhibitory concentration; MIC: elevated MIC; R: resistance; FCZ-R: fluconazole resistance; LMICs: low- and middle-income countries; HICs: high-income countries; LA: Latin America; BSI: bloodstream infection; NR: not reported; D28/D30: day 28/day 30 (mortality follow-up); CPC: clinical practice guideline.

## Data Availability

No new data were created or analyzed in this study. Data sharing is not applicable to this article.
